# The influence of *PER3* VNTR genotypes on the age of onset in a group of bipolar I disorder patients: an exploratory study

**DOI:** 10.1186/s40345-024-00346-7

**Published:** 2024-07-11

**Authors:** Tommaso Barlattani, Bettina Soltmann, Chiara D’Amelio, Valentina Socci, Francesca Pacitti, Maurizio Pompili, Philipp Ritter

**Affiliations:** 1 Chair of Psychiatry, Department of Biotechnological and Applied Clinical Sciences (DISCAB), University of L’Aquila, Via Vetoio, Coppito 2, L’Aquila, 67100 Italy; 2 Department of Psychiatry and Psychotherapy, University Hospital Carl Gustav Carus, Technische Universität Dresden, Dresden, Germany; 3 Department of Neuroscience, Mental Health, and Sensory Organs (NESMOS), Faculty of Medicine and Psychology, Unit of Psychiatry, Sapienza University, Sant’Andrea University Hospital, Rome, Italy

**Keywords:** Bipolar disorder, Age of onset, PER3 VNTR, Kaplan-Meyer, Melanopsin

## Abstract

**Background:**

*PER3* is a circadian gene that contains a variable number of tandem repeats (VNTR) which codifies for three genotypes: 4/4; 4/5; and 5/5 and is involved in non-visual response to light, a critical process associated with bipolar disorder onset. Benedetti et al. (Neurosci Lett 445(2):184–7) related this VNTR with bipolar disorder age of onset and linked genotype 5/5 with an earlier onset. In this study, we aimed to investigate these associations of PER3 VNTR genotypes with age of onset in a homogenous sample of German patients with bipolar I disorder through Kaplan-Meier curves.

**Methods:**

45 patients were enrolled and divided into three groups according to *PER3* VNTR genotypes. Recognizing common biological features, we built a combined group of -5 allele carriers (4/5 + 5/5). As a primary outcome, Kaplan-Meier analysis was conducted to delineate the three genotypes’ influence on age of onset. The secondary Kaplan-Meier analysis aimed to evaluate the relation between the 4/4 homozygotes group and the combined group (4/5 + 5/5) with age of onset. Finally, we proceeded to compare groups through a Log Rank Test and performed an analysis of covariance (ANCOVA).

**Results:**

The Kaplan-Meier analysis with three separate genotypes didn’t replicate the findings of Benedetti’s study. The analysis comparing genotype 4/4 with the combined group showed the influence of *PER3* VNTR variants on the age of onset and relates genotype 4/4 to an earlier onset. ANCOVA between the combined and the 4/4 genotype groups, correlated genotype 4/4 with an increased number of depressive episodes.

**Conclusion:**

This study showed no significant effect of P*ER3* VNTR genotypes on the age of onset and in linking genotype *5/5* with an earlier onset age. Contrasting results may arise from intrinsic differences between the two studies but also shed light on hypothetically different levels of functioning of *PER3* VNTR genotypes in the context of bipolar pathology. Further studies will require bigger and more homogeneous clinical samples.

**Supplementary Information:**

The online version contains supplementary material available at 10.1186/s40345-024-00346-7.

## Background

The utility of the age of onset, namely the age at which the patient first met criteria for a mood episode (manic, hypomanic, depressive or mixed), as a parameter for a better definition and understanding of bipolar and related disorders has been widely discussed (Leboyer et al. 2005). Moreover, bipolar patients often experience subthreshold symptoms before the onset age of the disease (Van Meter et al. 2016). Furthermore, when approaching bipolar disorder’s age of onset, gene-related parameters have been proven of utmost importance. A study conducted by Bellivier et al. (2001) and then reproduced in different samples (Hamshere et al. 2009; Manchia et al. 2008) identified three different onset groups and a polygenic non-Mendelian transmission was suggested for the early onset disease (Grigoroiu-Serbanescu et al. 2001). An earlier onset has an impact on the course of the illness (Propper et al. 2015; Subramanian et al. 2018) and has been related to a positive family history of bipolar disorder (Post et al. 2014). However, the lack of a clear association between single nucleotide polymorphisms and the age of onset, even when approaching homogenous samples, such as early-onset patients, suggests that the development and course of the illness are thereby influenced by genetic and environmental factors (Kalman et al. 2019).

A study conducted by Bauer et al. in 2012 observed an inverse relationship between the greatest solar insolation in spring, depending on latitude, and the age of onset, hypothesizing the role of regulatory genes in patients’ response to light. Light is recognized as the strongest external zeitgeber capable of synchronizing our core clock, the suprachiasmatic nucleus (SCN) (Roenneberg et al. 2007). Thanks to the blue-light sensitive melanopsin photopigment, the internal photosensitive retinal ganglion cells (ipRGC) can process environmental light by modulating the non-visual response to light (Hattar et al. 2002). These cells project to the SCN providing signals that determine the harmonization of the circadian cycle with the external environment (Lax et al. 2019).

Bipolar disorder appears to be associated with circadian alterations and the role of a disrupted harmony in circadian rhythms, such as alterations in the sleep architecture (Ritter et al. 2015) or impaired melatonin secretion (Etain et al. 2012), has been documented in the context of the pathology. The strong association with eveningness (Mansour et al. 2005) and the finding of a circadian disruption even in drug-naïve patients (Melo et al. 2017), has contributed to the identification of these as characteristic traits of the pathology, rather than episode’s dependent alterations, thus implying a possible genetic explanation. Our circadian clock responds to external cues, while internal zeitgebers are represented by circadian genes, that are regulated endogenously with feedback-loops mechanisms (Abreu and Bragança 2015).

*PER3* is a circadian gene of the period family expressed in the SCN (Archer et al. 2018). In humans, *PER3* dimerizes with *PER1*, *PER2* and *CRY1*, *CRY2* proteins inhibiting the *CLOCK BMAL1* complex and contributing to the maintenance of circadian period length (Matsumura and Akashi 2019). Variants of *PER3* have been associated with bipolar disorder (Mansour et al. 2006; Nievergelt et al. 2006; Brasil Rocha et al. 2017). Its coding region presents a variable number of tandem repeats (VNTR) on exon 18 with a sequence of 54 base pairs repeated 4 or 5 times, identifying 3 distinct genotypes: homozygous 4/4, heterozygous 4/5, and homozygous 5/5 (Ebisawa et al. 2001). The association of *PER3* VNTR genotypes with morningness or eveningness and a pathological condition such as delayed sleep phase syndrome (DSPD) confirmed that the number of repeated regions could influence the phosphorylation levels, affecting the gene’s functioning (Archer et al. 2003). A study conducted by Leocadio-Miguel et al. (2018) showed how *PER3* VNTR variants could regulate sleep-wake behavior with differences that appear to be related to latitude. Noticeably, this polymorphism modulates non-visual responses to light (Chellappa et al. 2014) by influencing the alerting response, melatonin suppression (Chellappa et al. 2012) and cognitive properties (Vandewalle et al. 2011).

Variants of *PER3* have been associated with bipolar disorder (Mansour et al. 2006; Nievergelt et al. 2006; Brasil Rocha et al. 2017). In this context, the VNTR polymorphism of *PER3* might influence the white matter integrity (Bollettini et al. 2017), the postpartum onset of the disease (Dallaspezia et al. 2011), the antidepressant effect of sleep deprivation (Dallaspezia et al. 2016) and the age of onset of the disease (Benedetti et al. 2008; Karthikeyan et al. 2014). The research conducted by Benedetti investigated the impact of *PER3* VNTR polymorphisms on the age of onset in a group of 99 patients diagnosed with bipolar I disorder through a Kaplan-Meyer analysis. Results from Benedetti’s study indicated an association of *PER3* VNTR genotypes with the age of onset of bipolar disorder and correlated genotype 5/5 with an earlier onset. Several works (Alexander et al. 2015; Dai et al. 2011; Guess et al. 2009; Yamaguchi et al. 2015; Wirth et al. 2014; Drake et al. 2015; Zhu et al. 2005; Viena et al. 2016) considered 4/5 and 5/5 genotypes as a single entity, thus focusing on common biological features and possible differences associated with the presence of the − 5 allele.

With the present study, we aimed to confirm the suggested association of *PER3* VNTR genotypes with age of onset in an independent sample of 45 German patients diagnosed with bipolar I disorder. Our primary goal was to evaluate the age of onset, defined as the first episode in which the patient meets the criteria for an affective episode, about the three separate genotypes of *PER3* VNTR. Secondarily, we explored a potential relation between the 4/4 homozygotes group and a combined group of -5 allele, both in hetero- and homozygosis (4/5 + 5/5) and their influence on age of onset, to focus on differences associated exclusively with the presence of the − 5 allele. Other secondary aims focused on assessing the relationship between *PER3* VNTR variants with the age of symptoms onset, thus including subthreshold symptoms, the polarity of onset (i.e., depressive, hypo-/manic or mixed first mood episode) and, finally, the number of episodes and suicide attempts.

## Methods

### Participants

The sample included 45 patients (21 males, 24 females) diagnosed with bipolar I disorder according to DSM-V criteria, which were in a euthymic phase at the time of the study. The recruited patients were already selected as samples for two separate chronobiology studies published elsewhere (Ritter et al. 2019, 2020).

The study was approved by the institutional review board of the Medical Faculty of the Technische Universität Dresden (IRB00001473 and IORG0001076).

We enrolled patients with bipolar I disorder from the outpatient departments of 4 different hospitals in Dresden and the nearby area, as well as by advertisement in local newspapers and websites of bipolar advocacy groups between 2016/2017. The age range was restricted to patients aged between 18 and 60 years of age. The study was conducted during the winter months (November 2015 to March 2016; November 2016 to March 2017) at latitude 51°N.

Written informed consent was obtained from all patients willing to participate in the study after a detailed description of the study’s aim and procedures. An experienced psychiatrist from our local psychiatric department assessed, employing accurate procedures, age of onset, age of first symptoms manifestation (including subthreshold symptomatology), number of depressive/manic/hypomanic or mixed episodes, number of manic episodes with psychotic features, hospitalizations, number of compulsory treatment or forced admission and the presence, and eventually the number, of suicide attempts. Age of onset was defined as the first episode that met the criteria for a hypo- /-manic, mixed or depressive episode as defined by DSM-V.

According to previous research underlining common biological features associated with the presence of the − 5 allele (Alexander et al. 2015; Dai et al. 2011; Guess et al. 2009; Yamaguchi et al. 2015; Wirth et al. 2014; Drake et al. 2015; Zhu et al. 2005; Viena et al. 2016), we built a combined group of -5 allele hetero- and homozygotes (4/5 + 5/5). Finally, to clarify the effect of 4/4 homozygotes and a combined group of -5 allele hetero- and homozygotes (4/5 + 5/5) on the age of onset, we performed a Kaplan-Meier analysis.

### Laboratory procedures

A blood sample was collected in 9 mL clotting tubes containing EDTA (S-Monovette K3, Sarstedt) for the genetic analyses. After centrifugation of the clotting tubes at 1000 g for 15 minutes, the EDTA whole blood was stored at − 80°C until the analyses were performed. Genomic DNA was extracted from frozen (− 80°C) EDTA whole blood using a QIAmp DNA blood mini kit (Qiagen) according to the manufacturer’s protocol. To quantify and assess the purity of the DNA, the absorbance at 260 nm and the ratios A 260/280 and A 260/230 were measured using a NanoDrop © 2000 spectrophotometer (Thermo Fisher). Polymerase chain reaction (PCR) was then performed with the following primers: 5’TGTCTTTTCATGTGCCCTTACTT-3’ and 5’TGTCTGGCATTGGAGTTTGA-3’. The PCR reaction was carried out in a 25 µL volume containing 150 ng DNA, 0.5 µM of each primer, 200 µM deoxynucleoside triphosphate, 1 × Q5 reaction buffer and 0.02 U/µL Q5 high-fidelity DNA polymerase (New England Biolabs). After an initial step of 30 s at 98 °C, 30 cycles of amplification (10 s at 98 °C, 30 s at 65 °C, 25 s at 72 °C) and a final elongation step of 5 min at 72 °C were performed. An aliquot of PCR product was analyzed on agarose (2% agarose LE, Biozym) gel electrophoresis (5 V/cm for 90 min) to distinguish between the − 5 repeat allele (401 bp), the 4 repeat allele (347 bp) and the heterozygotes (347 and 401 bp).

### Statistical analysis

We built cross tables for the description of sociodemographic and clinical data and then proceeded to calculate mean values, standard deviation, and frequencies for each variable considered. A chi-square test was conducted to evaluate whether the sample was in Hardy-Weinberg equilibrium. As the primary outcome, Kaplan-Meier analysis was performed to assess the potential influence of the three genotype subgroups (*PER3* 4/4; *PER3* 4/5; *PER3* 5/5) on the age of onset.

Analysis was carried out on three curves each for the single genotypes considered and then comparison between the groups was conducted through a Log Rank Test.

As a secondary outcome, according to previously demonstrated common biological features, we combined groups of *-5* allele hetero- and homozygotes (4/5 + 5/5). Aiming to clarify the effect of 4/4 homozygotes and the combined group of -5 allele in hetero- and homozygosis (4/5 + 5/5) on the age of onset, we performed a Kaplan Meier analysis. We constructed two curves for the two groups. We then proceeded to perform a Log Rank Test to compare the two groups.

Kaplan-Meier analysis was conducted for the age of symptomatic onset including subthreshold symptoms, age at the first depressive episode and age at first hypo- /-manic episode concerning the aforementioned groups, both separate and combined. Groups were compared through a Log Rank test for all the analyses mentioned.

Finally, we carried out univariate analysis of covariance ANCOVA for group comparisons of different variants such as number of depressive, manic (manic plus hypomanic) and mixed episodes, number of depressive and manic episodes with psychotic symptoms, number of inpatient stays, number of forced admissions or compulsory treatments, and number of suicide attempts, all of which were adjusted for the duration of the illness. The analysis included age at onset as a covariate. For the analyses carried out, we defined statistical significance as demonstrated by a P-value of less than 0.05. Statistical analysis was conducted using IBM SPSS statistics for Windows, version 26.

## Results

### Primary outcome: age of onset

The average age of onset was (mean ± standard deviation) 21,84 ± 9,322.

Observed genotype frequencies were: *PER3* 4/4 17/45 (37.8%), *PER3* 4/5 21/45 (46.7%), *PER3* 5/5 7/45 (15.5%). The sample was in Hardy-Weinberg equilibrium as confirmed by the Chi-square test (χ2 = 0.15, *p* = 0.993).

**Fig. 1:**
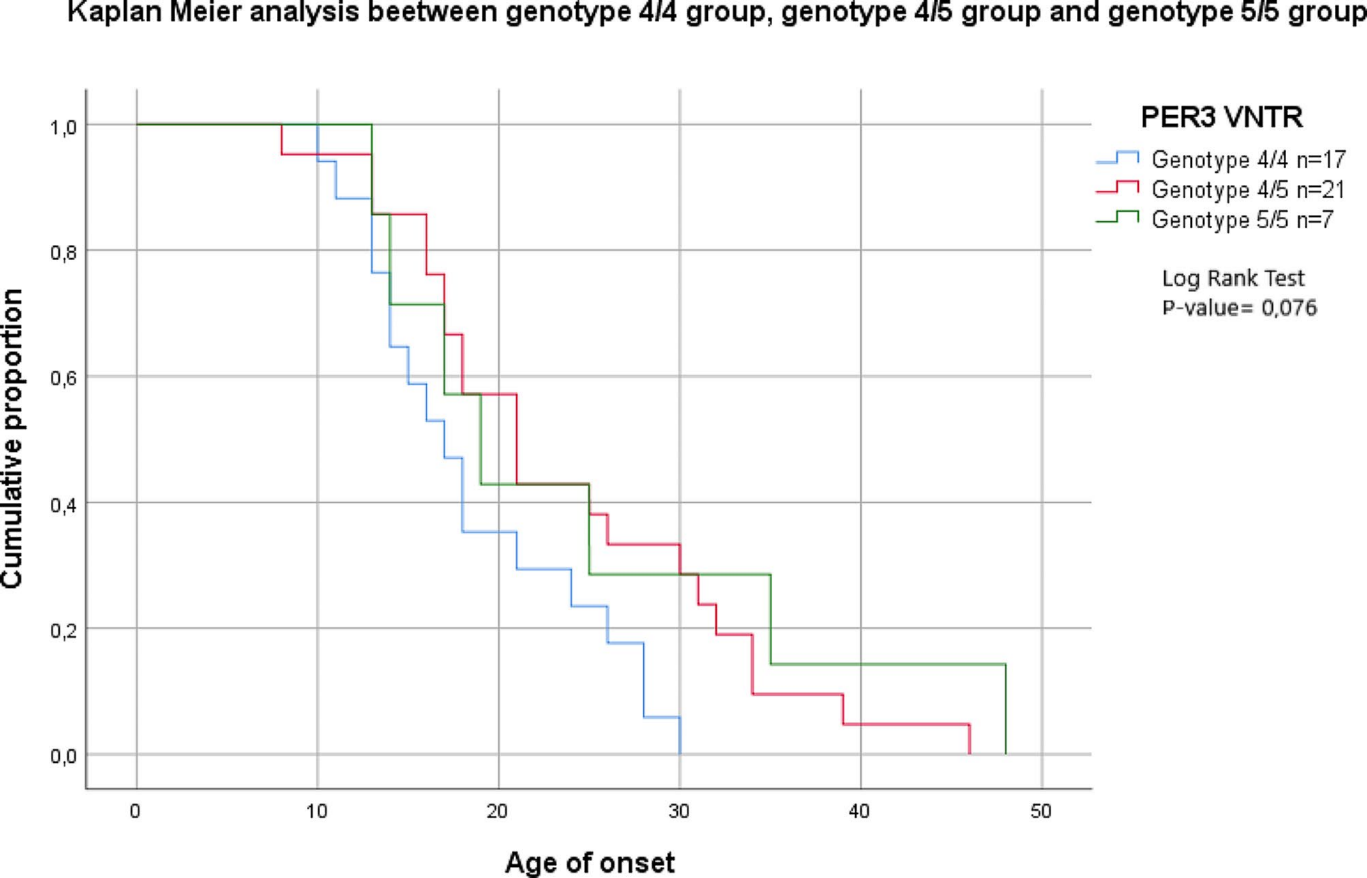
The lifetime distribution of age at onset in the three genotypic groups

The Kaplan-Meier analysis was conducted to determine the influence of *PER3* VNTR variants on the age of onset for the three separate genotypes. Although 5/5 homozygotes presented a later age of onset when compared to both heterozygotes and 4/4 homozygotes, reported differences were not statistically significant (Fig. 1; Table 1).

**Fig. 2:**
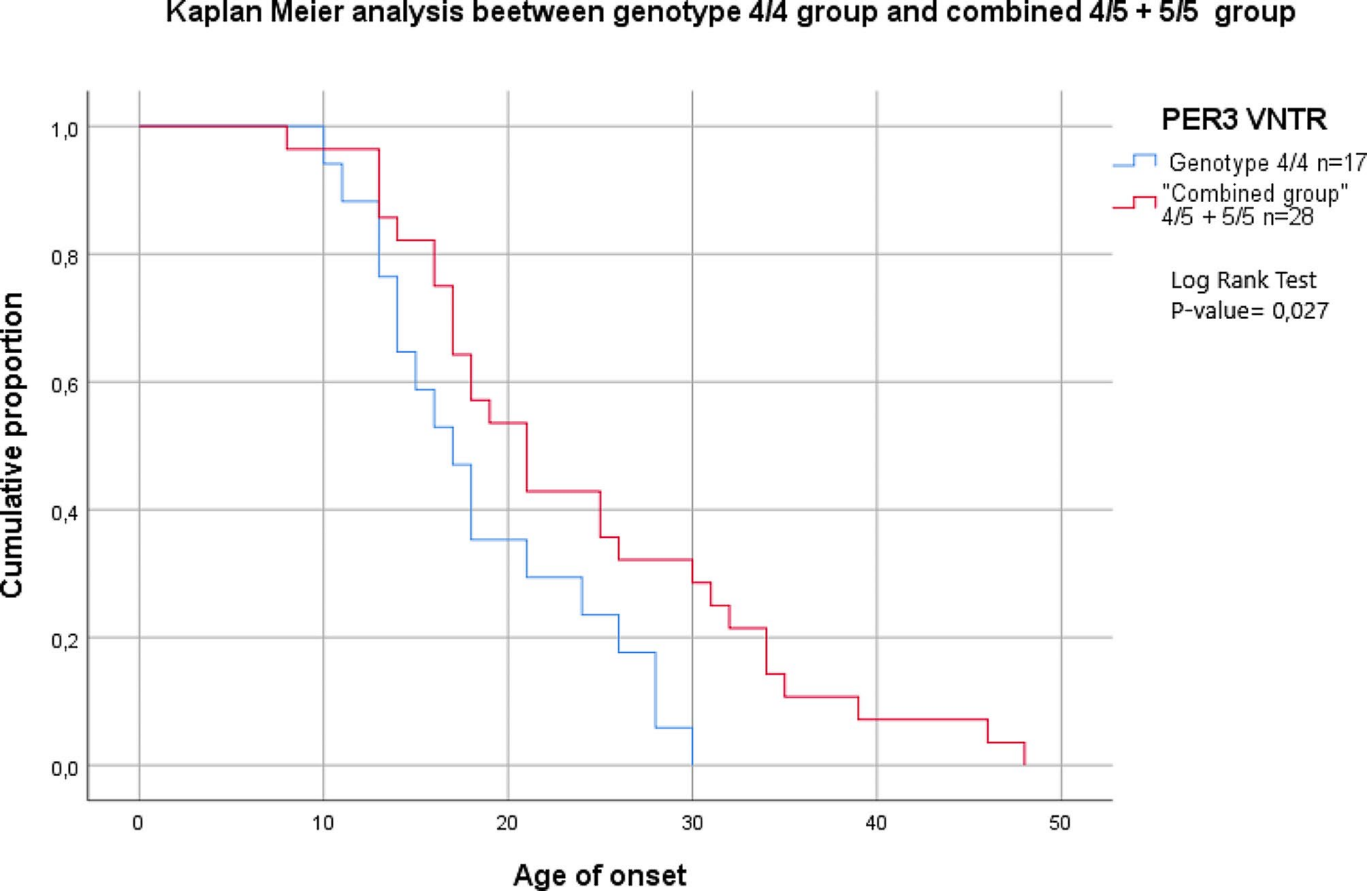
The lifetime distribution of age at onset in the genotype *4/4* group and the *4/5* + *5/5* combined group

Results of the Kaplan-Meier analysis, performed for the 4/4 and the combined group of 4/5 and 5/5 genotypes, demonstrated an influence of both groups on the age of onset, namely an association with an earlier onset, as demonstrated by an average age of 18,588 ? 1,55, for the 4/4 homozygotes and an average age of 23,821 ? 1,95 for the combined group confirmed by statistical evidence, P-value= 0,027, in the Log Rank Test (Fig. 2; Table 2).

**Table 1:** Age of onset according to PER3 VNTR three genotypes

	PER3 VNTR			Log Rank Test
	*4/4* (*N* = 17)	*4/5* (*n* = 21)	*5/5* (*n* = 7)	*P*-value
Age of onset (mean ± SD)	18,588 ± 1,55	23,619 ± 2,12	24,429 ± 4,86	0,076
Age of first symptoms (mean ± SD)	18,353 ± 1,52	21,905 ± 2,05	25,714 ± 4,71	0,130
Age of the first depressive episode (mean ± SD)	19,467 ± 1,47	23,650 ± 2,32	24,571 ± 1,42	0,219
Age of the first hypo-/-manic episode (mean ± SD)	24,059 ± 1,99	27,857 ± 2,04	27,000 ± 4,98	0,395

*AbbreviationsVNTR*: Variable number tandem repeats; *SD*: Standard Deviation

**Table 2:** Age of onset according to PER3 VNTR 4/4 group and combined group 4/5 + 5/5

	PER3 VNTR		Log Rank Test
	*4/4* (*n* = 17)	*4/5 + 5/5* (*n* = 28)	*P*-value
Age of onset (mean ± SD)	18,588 ± 1,55	23,821 ± 1,95	0,027
Age of first symptoms (mean ± SD)	18,353 ± 1,52	22,857 ± 1,91	0,065
Age of the first depressive episode (mean ± SD)	0,219 ± 1,47	23,889 ± 2,09	0,092
Age of the first hypo- /-manic episode (mean ± SD)	24,059 ± 1,99	27,643 ± 1,92	0,173

*AbbreviationsVNTR*: Variable number tandem repeats; *SD*: Standard Deviation

### Secondary outcomes

The Kaplan-Meier analysis was conducted to determine the influence of *PER3* VNTR variants on the three separate genotypes regarding the age of symptomatic onset, the age of the first depressive episode and of the first manic (hypo- /-manic) episode, numerically 4/4 homozygosis was associated with an earlier onset when compared to both heterozygosis and 5/5 homozygotes, although reported differences that were not statistically significant (Supplementary Fig. 3; Supplementary Fig. 5; Supplementary Fig. 7).

Moreover, analyses carried out for the same parameters but comparing the combined group composed of 4/5 and 5/5 individuals with homozygous individuals for genotype 4/4 reported no statistical significance (Supplementary Fig. 4; Supplementary Fig. 6; Supplementary Fig. 8).

The ANCOVA analysis conducted on the three separate genotypes and adjusted for the duration of illness and with the age of onset as a covariate, showed no statistically significant association with the total amount of depressive episodes, manic (hypo- /-manic) episodes, mixed episodes, depressive episodes with psychotic symptoms, manic episodes with psychotic symptoms, number of stationary phases, number of suicide attempts and of inpatient stays with none of the genotype taken into account (Supplementary Table 4).

Furthermore, ANCOVA analysis adjusted for the duration of illness and with the age of onset as a covariate, conducted to compare the 4/4 genotype group and the combined 4/5–5/5 group, revealed no statistically significant association with the total amount of manic (hypo- /-manic) episodes, mixed episodes, depressive episodes with psychotic symptoms, manic episodes with psychotic symptoms, number of stationary phases, number of suicide attempts and of inpatient stays. Nevertheless, the analysis demonstrated a statistically significant higher number of depressive episodes linked to the 4/4 genotype group, with an average of 17,59 ± 23,86 episodes and P-value = 0,026 (Table 3).

**Table 3:** Episodes according to PER3 VNTR 4/4 group and combined group 4/5 + 5/5

	PER3 VNTR		ANCOVA
	*4/4* (*n* = 17)	*4/5* + *5/5* (*n* = 28)	*P*-value
Number of depressive episodes (mean ± SD)	17,59 ± 23,86	8,75 ± 6,73	0,026
Number of hypo- /- manic episodes (mean ± SD)	24,06 ± 8,24	27,64 ± 10,19	0,484
Number of mixed episodes (mean ± SD)	6,50 ± 14,65	3,50 ± 9,42	0,682
Number of manic episodes with psychotic symptoms (mean ± SD)	1,06 ± 2,02	1,21 ± 2,11	0,742
Number of depressive episodes with psychotic symptoms (mean ± SD)	1,71 ± 3,08	0,39 ± 0,79	0,107
Number of inpatient stays (mean ± SD)	1,47 ± 1,50	4,36 ± 4,70	0,058
Number of forced admissions (mean ± SD)	0,53 ± 1,18	0,79 ± 1,40	0,818
Number of suicide attempts (mean ± SD)	0,59 ± 1,18	0,64 ± 1,06	0,794

*AbbreviationsVNTR*: Variable number tandem repeats; *SD*: Standard Deviation

## Discussion

In the present study, the Kaplan-Meier analysis was conducted to investigate the association of *PER3* VNTR genotypes with age of onset in an independent sample of 45 patients diagnosed with bipolar I disorder. Results showed no significant effect of P*ER3* VNTR genotypes on the age of onset and in linking genotype *5/5* with an earlier onset age as demonstrated in Benedetti’s previous study.

Despite those differences that could occur by chance, some considerations need to be made.

The first consideration concerns differences between methodologies employed in the two studies for the statistical analysis. Benedetti’s group used a two-way ANOVA, with sex and genotype as co-factors, while in our study, curves of Kaplan-Meier’s analysis were compared using the Log Rank test. Another consideration regards differences in characteristics of the groups considered. Particularly, dissimilarities between the two groups are mostly prominent when focusing on the age of onset. Benedetti’s sample presented the age of onset comparable to an intermediate onset (mean age of onset 31,78 ± 11,18), while our sample’s age of onset (mean 21,84 ± 9,322) was more congruent with the average of bipolar I disorder, which is around 22 years of age (Goodwin and Jamison 2007). This result could not be justified by a delayed age of onset related to differences in average age between Italy and Germany. Moreover, as previously shown by the study of Bauer et al. (2012), the amount of sunlight exposure is also an important factor whose effect on the onset of the disease must be considered, therefore differences in latitudes between the two countries may be accountable for such discrepancy. But, despite the city of Milan, where Benedetti’s study was conducted, being at a latitude of 45° and the city of Dresden is at a latitude of 51°, the difference in slope in a change of solar insolation is unlikely to be substantially different between the two cities. Thus, differences in latitude are probably not accountable for such a delayed onset, which should be therefore regarded as an intrinsic characteristic of the examined group. Differences in capturing this parameter, with such a late onset for Benedetti`s sample could have led to skewed results.

As a result of the comparison between the 4/4 genotype group and the combined group presenting the − 5 allele, we observed the influence of *PER3* VNTR variants on the age of onset. However, while results from Benedetti’s study from 2008 demonstrated an association of the *PER3* 5/5 variant with an earlier onset, data obtained from our study revealed a substantially opposite association. In our study, we found genotype 4/4 to be more commonly associated with an earlier onset of disease. Noticeably, other studies on *PER3* VNTR variants yielded such conflicting results. The study carried out by Pereira et al. (2005) correlated DSPD with *PER3* VNTR genotypes, resulting in radical opposition with previous studies, but in line with our findings as, in the morning immediately after sleep, subjects exposed to blue light with genotype 4/4 exhibit substantially higher activation of parietal and prefrontal areas (Vandewalle et al. 2011). This enhanced response is also supported by evidence of a greater response to the application of supplementary light in the blue wavelength during this part of the day (Turco et al., 2017). The role of morning light in bipolar disorder has been already established when approaching therapeutic options (Benedetti et al., 2001), but may also be significant when focusing on the onset of the disorder in genotypically more light-sensitive subjects during that particular time of the day. Most drastic changes in light conditions take place during dusk and dawn when the light reflects the blue wavelength (Foster 2005; Dick 2012), these changes are most prominent also during changing of seasons and are also strictly dependent on latitude. Indeed, the sudden fluctuations related to the change of season and to the different light phases during the day affect the subjects carrying these genes more intensely, especially in the morning. Despite the hypothetical character of our observations, it is legitimate to suppose that the conflicting results presented in this study may also derive from a complex interrelation between genetic factors and environmental conditions.

Focusing on the number of depressive episodes, comparing genotype 4/4 with the combined group composed of genotype 4/5 and 5/5, we obtained a statistical analysis of significant value. These results are in line with previous studies that linked a depressive onset, a tendency in our study for 4/4 genotype carriers and not sustained by statistical evidence, with a higher number of depressive episodes (Daban et al. 2006).

The major limitations of our study rely on the limited number of subjects taken into consideration, along with the relative reliability of the age of onset parameter, since patients may often not recollect precisely the age of the disease’s manifestation. Further studies may need samples larger and more homogeneous in terms of both samples and methods of analysis. Finally, multicenter studies could clarify whether effective environmental factors influence *PER3* VNTR genotype functions in the context of bipolar disorder regarding age of onset.

### Electronic supplementary material

Below is the link to the electronic supplementary material.

Supplementary Material 1

## Data Availability

The datasets used and/or analysed during the current study are available from the corresponding author on reasonable request.

## Abbreviations

VNTR: Variable number of tandem repeats
ANCOVA: Analysis of covariance
SCN: Suprachiasmatic nucleus
ipRGC: internal photosensitive retinal ganglion cells
DSPD: Delayed sleep phase disorder
