# Supplementary material for: The influence of PER3 VNTR genotypes on the age of onset in a group of bipolar I disorder patients: an exploratory study
Source: Int J Bipolar Disord. 2024 Jul 11;12:25. doi: 10.1186/s40345-024-00346-7 (PMC11239620; doi:10.1186/s40345-024-00346-7)
Supplement: Supplementary file 1 — Supplementary Material 1 [file 40345_2024_346_MOESM1_ESM.docx]

**Supplementary information**

|  | *PER3* VNTR | | | ANCOVA |
| --- | --- | --- | --- | --- |
|  | *4/4* (n=17) | *4/5* (n=21) | *5/5* (n=7) | P-value |
| Number of depressive episodes (mean ± SD) | 17,59 ± 23,86 | 8,00 ± 6,20 | 11,00 ± 8,25 | 0,064 |
| Number of hypo-/-manic episodes (mean ± SD) | 24,06 ± 8,24 | 27,86 ± 9,37 | 27,00 ± 13,17 | 0,689 |
| Number of mixed episodes (mean ± SD) | 6,50 ± 14,65 | 4,52 ± 10,73 | 0,43 ± 1,13 | 0,694 |
| Number of manic episodes with psychotic symptoms (mean ± SD) | 1,06 ± 2,06 | 1,14 ± 2,33 | 1,43 ± 1,40 | 0,866 |
| Number of depressive episodes with psychotic symptoms (mean ± SD) | 1,71 ± 3,08 | 0,43 ± 0,81 | 0,29 ± 0,76 | 0,218 |
| Number of inpatient stays (mean ± SD) | 1,47 ± 1,50 | 4,67 ± 5,20 | 3,43 ± 2,76 | 0,106 |
| Number of forced admissions (mean ± SD) | 0,53 ± 1,18 | 0,86 ± 1,53 | 0,57 ± 0,79 | 0,885 |
| Number of suicide attempts (mean ± SD) | 0,59 ± 1,18 | 0,67 ± 1,07 | 0,57 ± 1,13 | 0,924 |

***Supplementary Table 4*.** Episodes according to *PER3* VNTR three genotypes

**Abbreviation**: VNTR: Variable number tandem repeats; SD: Standard Deviation

**
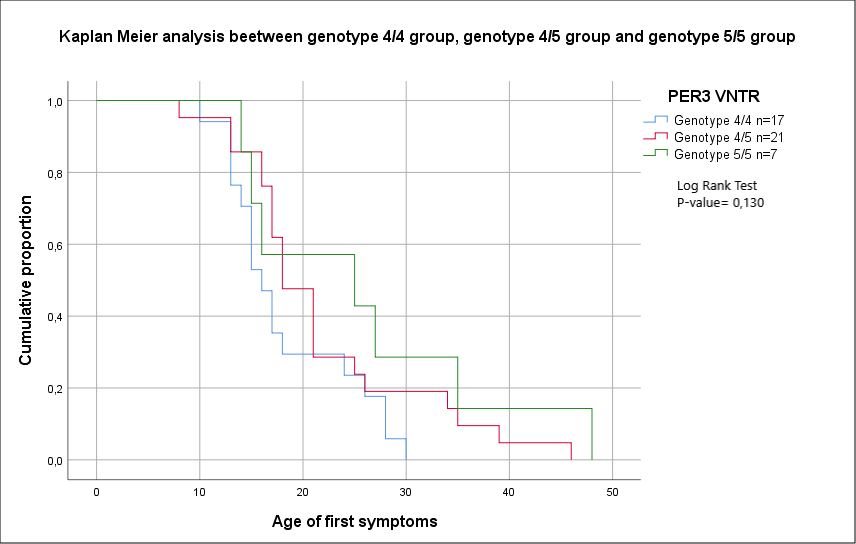
**

***Supplementary Figure 3*. The lifetime distribution of age of the first symptoms in the three genotypic groups**

**
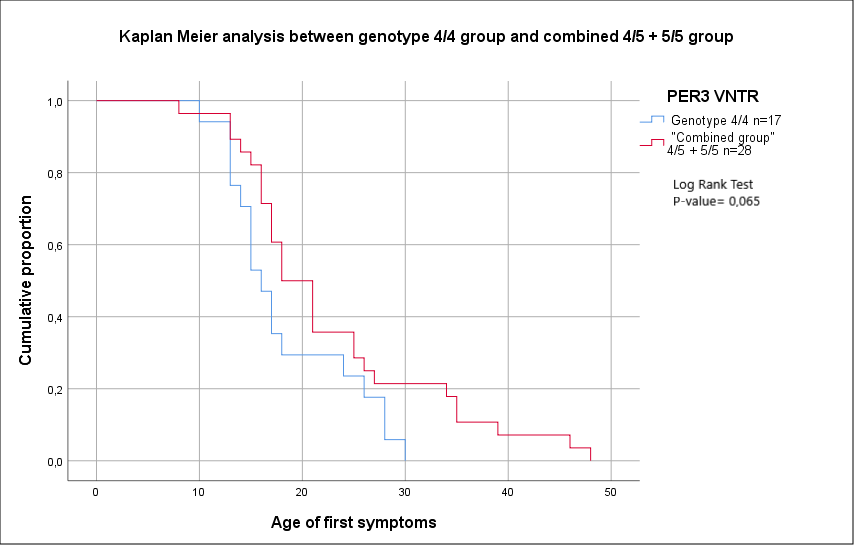
**

***Supplementary Figure 4*. The lifetime distribution of age of the first symptoms in the genotype *4/4* group and in the combined *4/5* + *5/5* group**

**
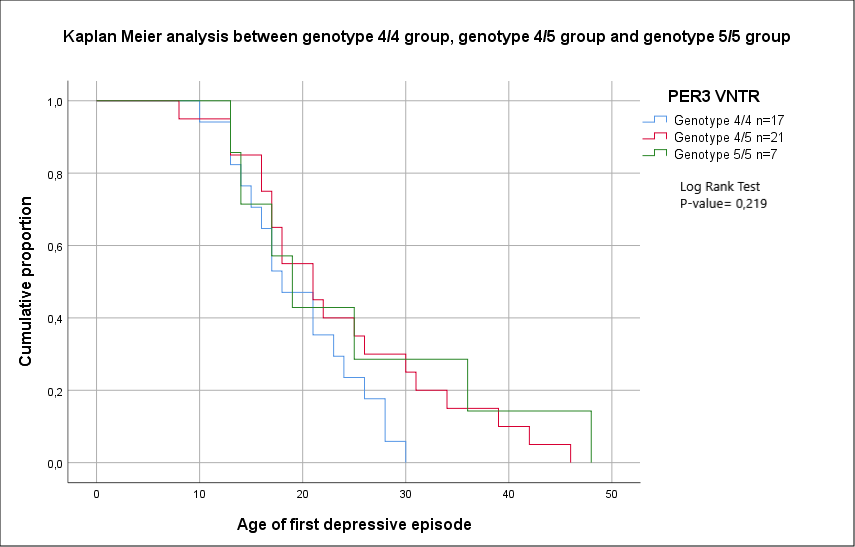
**

***Supplementary Figure 5*. The lifetime distribution of age of the first depressive episode in the three genotypic groups**

**
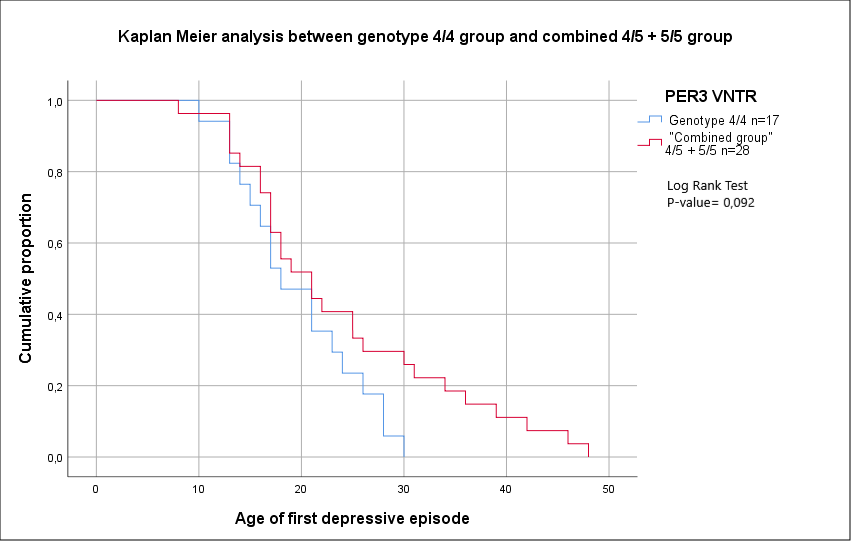
**

***Supplementary Figure 6*. The lifetime distribution of age of the first depressive episode in the genotype *4/4* group and the combined *4/5* + *5/5* group**


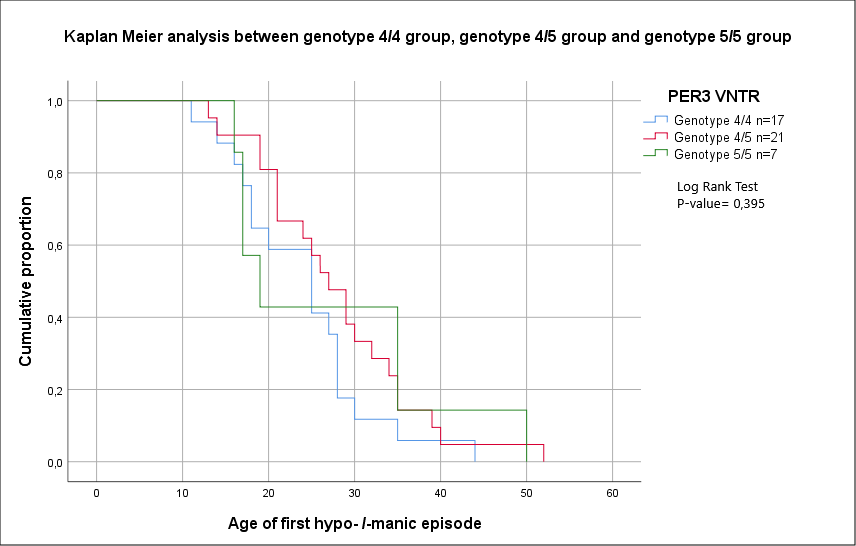


***Supplementary Figure 7*. The lifetime distribution of age of the first hypo- /-manic episode in the three genotypic groups**

**
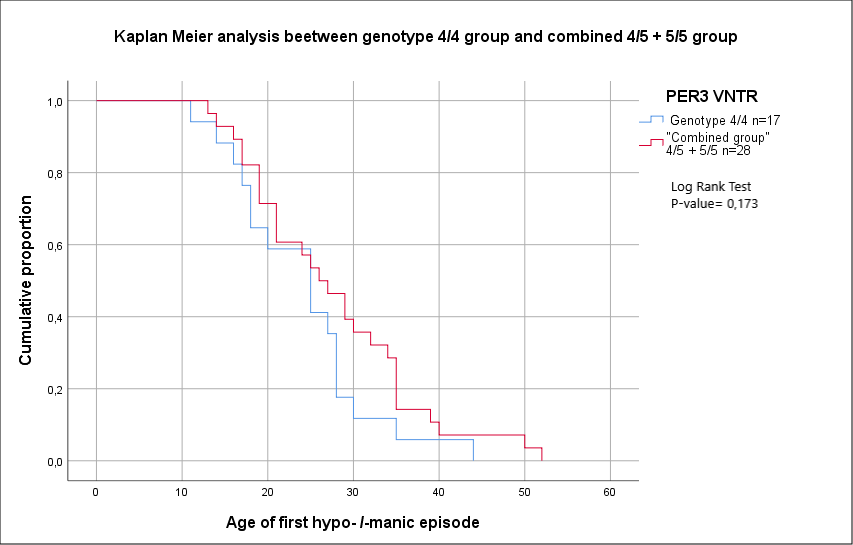
**

***Supplementary Figure 8*. The lifetime distribution of age of the first hypo- /-manic episode in the genotype *4/4* group and the combined *4/5* + *5/5* group**
